# The effect of environmental factors on precocious puberty in children: a case–control study

**DOI:** 10.1186/s12887-023-04013-1

**Published:** 2023-05-01

**Authors:** Francis Manyori Bigambo, Dandan Wang, Qing Niu, Mingzhi Zhang, Sabitina Mrisho Mzava, Yubing Wang, Xu Wang

**Affiliations:** 1 School of Public Health, Nanjing Medical University, Nanjing, 211166 China; 2 Department of Endocrinology, Children’s Hospital of Nanjing Medical University, 72 Guangzhou Rd, Nanjing, 210008 China; 3 Muhimbili National Hospital, P. O. Box 65000, Dar Es Salaam, Tanzania

**Keywords:** Precocious puberty, Cesarean section, Secondhand smoke, Child BMI, Adequate sleep, Children

## Abstract

**Background:**

Recently the prevalence of precocious puberty development is increasing among Chinese children. Proper understanding of the risk factors for precocious puberty in children is pivotal as could help to improve children's health. This study aims to evaluate the effect of environmental factors on precocious puberty in children.

**Methods:**

We matched the cases and controls by age at the ratio of 1:1 (201 cases and 201 controls) for girls and 1:4 (24 cases and 96 controls) for boys. We used conditional logistic regression to explore the effect of environmental factors on precocious puberty, and a random forest model to identify the most important risk factor.

**Results:**

In the multivariate regression, cesarean section (OR = 1.99, 95% CI: 1.05, 3.76), child body mass index [BMI] (OR = 1.25, 95% CI: 1.10, 1.43), maternal BMI (OR = 1.13, 95%CI: 1.01, 1.26), and exposure to secondhand smoke several times a month but less than once a week (OR = 4.09, 95%CI: 1.79,9.35), and almost every day (OR = 6.48, 95% CI: 2.14, 19.56) were risk factors for precocious puberty in girls. While maternal height (OR = 0.82, 95% CI: 0.75, 0.88), paternal height (OR = 0.91, 95% CI: 0.85, 0.98), bedtime at night (OR = 0.30, 95% CI: 0.17, 0.51), and night sleep (OR = 0.43, 95% CI: 0.21, 0.86) were protective factors. In boys, only exposure to secondhand smoke several times a month but less than once a week (OR = 7.94, 95% CI: 1.25, 50.33) was a risk factor for precocious puberty. In the random forest model, Child BMI was the most important risk factor for precocious puberty in girls.

**Conclusions:**

Our findings suggest that environmental factors were associated with precocious puberty in children, particularly in girls.

**Supplementary Information:**

The online version contains supplementary material available at 10.1186/s12887-023-04013-1.

## Background

Puberty is a complex biological and psychological change from childhood to adulthood, which usually began at ages 8–13 years in girls and 9–14 years in boys [1]. While precocious puberty is early onset of puberty with the development of secondary sexual characteristics before the age of 8 in girls and 9 years in boys [2]. The prevalence of precocious puberty is increasing among Chinese children. A school population-based study showed that the percentage of girls and boys with signs of precocious puberty before 8 and 9 years was 11.47% and 3.26%, respectively [3]. The reasons for the increase in the trends of precocious puberty onset among these children remain unclear. However, it is plausible that the incidence of precocious puberty onset is increasing with the improvement of living standards and the influences of environmental factors [4–6].

Epidemiological studies have reported controversial results about the factors associated with pubertal development in children. Proper understanding of the factors associated with precocious puberty is pivotal as could help to improve the preventive strategies for precocious puberty development, and hence reduce children’s chance of having precocious puberty and related adverse health outcomes.

After the outbreak of the novel COVID-19, China and other countries globally enacted lockdown policies as the best method to reduce the rate of spread of COVID-19 infection. During the lockdown, unhealthy lifestyles and behaviors were observed including lack of control around food, over-eating or skipping meals, sleep problems, decreased physical activity and changes in body weight [7–9], increased use of screening devices, smoking behavior, and alcohol consumption [9]. In addition, during the pandemic, the consultation of suspected cases of precocious puberty was reported to be increased. For example, in Brazil, the consultation for suspected cases of precocious puberty increased by 50% during the COVID-19 pandemic [10]. In Italy, during and after the lockdown, the incidence of girls diagnosed to have central precocious puberty (CPP) was higher than in the previous 5 years [11]. Similarly, a study from India observed an increase in both referrals for precocious puberty and children diagnosed to have idiopathic central precocious puberty (ICPP) during lockdown [12]. We hypothesized that environmental factors as the result of the lockdown may also contribute to the development of precocious puberty. Indeed, an increasing body of evidence has emerged that precocious puberty is influenced by environmental factors including social demographic factors such as sex (being a girl), age (girl of 7 years), better economic development [4], premature birth [13], and cesarean delivery in boys [14]. In addition, other environmental factors associated with precocious puberty in children include dietary habits such as formula feeding after birth [15], diets heavy in desserts/snacks, soft drinks, and fried food without adjustment for social-economic factors [16]; anthropometric measurements such as maternal obesity and childhood BMI [17, 18]; sleep habits such as delayed sleep and disrupted sleep [19] and; parenting habits include allowing children to stare on-screen devices such as television or computer [20, 21] and secondhand smoke [21]. However, some relevant studies have also reported inconsistent results [16, 22, 23].

To bridge these research gaps, we conducted a matched case–control study to identify the effect of environmental factors on precocious puberty in children who attended the Department of Endocrinology of Children's Hospital of Nanjing Medical University in China.

## Methods

### Participants and study design

In the present study, children diagnosed to have precocious puberty in the department of Endocrinology of Nanjing Children's Hospital in China from April 2021 to September 2021 were included in the case group, and normal children (without precocious puberty) were matched according to age in the control group. The inclusion criteria for the case group were as follows: 1) Children conforming to the diagnostic criteria for precocious puberty, which is secondary sexual characteristics development in advance before 8 years of age in girls and 9 years in boys. 2) Children and their guardians (caregivers) agreed to participate and signed informed consent. Exclusion criteria include; 1) Secondary central precocities, such as central nervous system occupying, infection, trauma, postoperative, radiotherapy or chemotherapy, and congenital dysplasia. In addition, other primary diseases that may lead to precocious puberty, such as congenital adrenal hyperplasia, McCune-Albright syndrome, and precocious puberty associated with congenital hypothyroidism. 2) Children and their guardians who did not agree to participate in the study.

The inclusion criteria for the control group were as follows: 1) Normal healthy children not conforming to the diagnostic criteria for precocious puberty. 2) Children and their caregivers agreed to participate and signed informed consent.

**Fig. 1:**
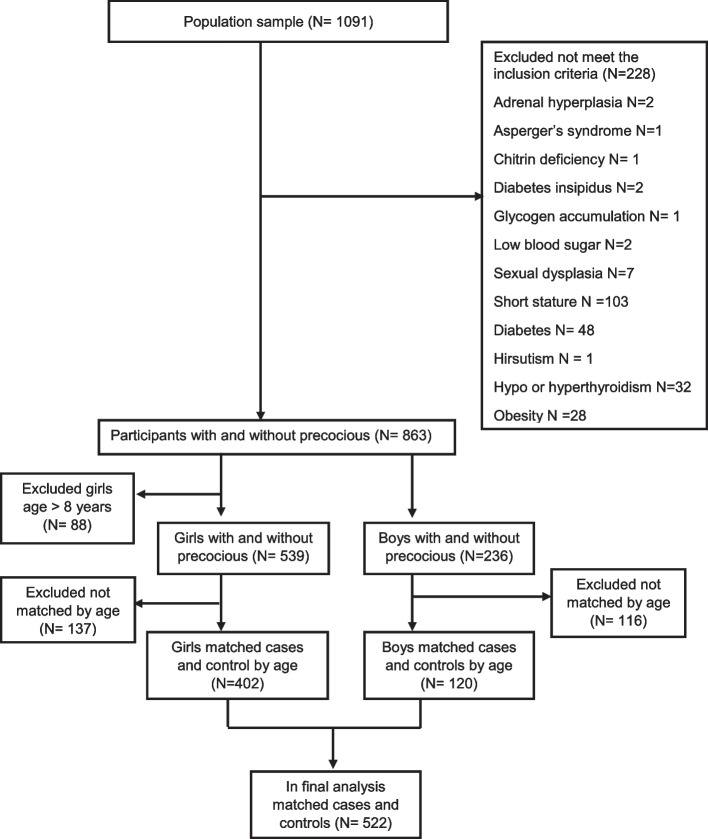
Flow diagram of the selection process of the study participants

Among 1091 children who agreed to participate in the present study, 228 children were excluded because they did not meet the inclusion criteria. We also excluded 88 girls aged above 8 years old. The participants who remained in the case–control study were 775 children, among them, 539 (338 cases and 201 controls) were girls and boys were 236 (24 cases and 212 controls). We matched the cases and controls by age separately for girls and boys at the ratio of 1:1 (201 cases and 201 controls) for girls and 1:4 (24 cases and 96 controls) for boys. Participants remaining, in the final analysis, were 522 (Fig. 1). The research content and informed consent were approved and confirmed by the Institutional Review Board of Children's Hospital of Nanjing Medical University according to the requirements of relevant laws and regulations. All children’s caregivers were given written informed consent.

### Data collection methods

We obtained information about the study participants by using a structured questionnaire. The investigator filled out the questionnaires during a face-to-face interview with the children’s caregivers. The questionnaire contains five sections including 1) Children’s general information such as age, race/ ethnicity, resident, caregiver education background, parity, delivery method, and preterm birth. 2) Anthropometric measurements of children include height, weight, body mass index (BMI), maternal height, maternal weight, maternal BMI, paternal height, paternal weight, paternal BMI, maternal history of early puberty, and paternal history of puberty. Children’s height and weight were measured while children dressed lightly and barefoot. 3) Diet habits of children including; feeding method after birth by asking “How is your child fed after birth?”, barbecued food by asking “Does your child like barbecued food?”, eating dessert by asking “Does your child like cake, chocolate, ice cream and other desserts?”, soft drinks by asking “Does your child like soft drinks such as soda, coke, juice, milk tea, etc.?”, receiving vitamin D by asking “Has your child taken vitamin D (cod liver oil) supplements since birth?”, and children’s bowl material used by asking “Which of the following materials your child’s bowl made of ?”. 4) Sleep habits including; average bedtime at night by asking “At what time does your child usually go to bed during the night?”, average sleep hours per day (24 h) by asking “How many hours in average does your child sleep per day (24 h.)?”, average day sleep from 07:00–19:00 (hrs.) by asking “How many hours in average does your child sleep from 07:00 AM to 19:00 PM?”, night sleep from 19:00–07:00 (hrs.) by asking “How many hours in average does your child sleep from 19:00 PM to 07:00 AM?”, wake up at night by asking “How many times a day on average does your child wake up during the night?”, time caregiver spends to put a child to sleep by asking “How long do you usually take to put your child to sleep at night?”, and child snore by asking “Does your child snore in his/her sleep?”. 5) Parenting habits include the child’s time staring at screening devices by asking “How long does a child watch TV or computer, cell phone, and other electronic devices every day?”, the child’s time spent outdoors by asking “What is the average time your child spends in outdoors on sunny days?”, rate of outdoor activities by asking “What is the intensity of your child outdoor activities?”, and secondhand smoke by asking “Does anyone in your child’s environment smoke?”. In this study, secondhand smoke referred to smoke exhaled into the environment by a smoker following inhalation of cigarette smoke or smoke released from burning of the tip of cigarette or tobacco products [24]. To ensure the quality of clinical research practice, the investigators were trained before the data collection process.

### Statistical analysis

We presented frequencies and percentages for categorical variables and mean ± Standard deviation (SD) for continuous variables. We used the Pearson chi-square test (χ^2^) or Fisher’s exact test to identify variables with significant differences between case and control groups for categorical variables and the student’s t-test for continuous variables. We performed univariate and multivariate conditional logistic regressions to evaluate the effect of environmental factors on precocious puberty in children. All the variables with significant differences between the case and control groups at *p* < 0.05 in either girls or boys were incorporated in the univariate conditional logistic regression analyses. In the multivariate conditional logistic regression, we included all the variables that were identified to be statistically significant risk factors for precocious puberty in children from the univariate regressions. All the analyses were performed separately for girls and boys by using Stata software version 15 (StataCorp LLC, USA). A statistical significance was considered at a two-sided *p* < 0.05.

We also performed a random forest algorithm to evaluate the level of importance of the factors associated with precocious puberty obtained from the multivariate conditional logistic regression in girls. We sampled the participants into the train set (80%) and test set (20%). We used a train set to construct 501 trees incorporating all the risk factors that were statistically significant from the multivariate conditional logistic regression. At each split, potential predictor variables were randomly selected. Then we created a plot showing the level of importance of the selected potential predictor variables. Finally, we used a test set to evaluate the strength of the model by creating a receiver operating characteristic (ROC) curve and calculating the area under the curve (AUC). We conducted random forest in R version 4.0.1 by using the randomForest package 4.6–14.

## Results

Overall, 522 children participated in this study. In girls, cases were 201 and controls were 201. The mean ages of the participants were 7.20 ± 1.11 and 7.42 ± 0.49 years for cases and controls, respectively. Resident (*p* =  < 0.001, Fisher’s exact test), caregiver education (*p* =  < 0.001, Fisher’s exact test), and delivery method (χ^2 =^ 7.89, *p* = 0.005) were significant differences between the case and control groups. In boys, cases were 24 and controls were 96. The mean ages of the participants were 8.58 ± 2.41 and 7.47 ± 0.60 years for cases and controls, respectively. Resident (*p* = 0.001, Fisher’s exact test), caregiver education (*p* =  < 0.001, Fisher’s exact test), and preterm birth (χ^2 =^ 4.90, *p* = 0.027) were significant differences between the case and control groups (Table 1).

**Table 1:** General information for the case and control groups

Variable	Case	Control	t /χ^2^	*P*-value
**Girls**				
** N (%)**	201 (50)	201 (50)		
** Age (years)**	7.20 ± 1.11	7.42 ± 0.49	2.48	**0.007**
**Ethnicity (%)**			2.34	0.126
Han	196 (97.51)	190 (94.53)		
Others	5 (2.49)	11 (5.47)		
**Resident (%)**				< 0.001^*^
City	169 (84.08)	201 (100)		
Township	24 (11.94)	0 (0.00)		
Countryside	8 (3.98)	0 (0.00)		
**Caregiver education (%)**				< 0.001^*^
Elementary school and below	5 (2.49)	2 (1.00)		
Junior high school	14 (6.97)	2 (1.00)		
High school/technical secondary school	30 (14.93)	12 (5.97)		
College/vocational college	48 (23.88)	29 (14.43)		
University and above	104 (51.74)	156 (77.61)		
**Parity (%)**			0.09	0.770^*^
1	175 (87.06)	173 (86.07)		
2	26 (12.94)	28 (13.93)		
3	-	-		
**Delivery method (%)**			7.89	**0.005**
Natural birth	97 (48.26)	125 (62.44)		
Cesarean section	104 (51.74)	76 (37.56)		
**Preterm birth (%)**			0.88	0.347
Yes	26 (10.24)	20 (9.95)		
No	175 (89.76)	181 (90.05)		
**Boys**				
** N (%)**	24 (20)	96 (80)		
** Age (years)**	8.58 ± 2.41	7.47 ± 0.60	-4.08	1.000
**Ethnicity (%)**				0.626^*^
Han	22 (91.67)	91 (94.79)		
Others	2 (8.33)	5 (5.21)		
**Resident (%)**				0.001^*^
City	18 (75.00)	93 (96.88)		
Township	4 (16.67)	3 (3.13)		
Countryside	2 (8.33)	0 (0.00)		
**Caregiver education (%)**				< 0.001^*^
Elementary school and below	1 (4.17)	0 (0.00)		
Junior high school	3 (12.50)	3 (3.13)		
High school/technical secondary school	7 (29.17)	8 (8.33)		
College/vocational college	5 (20.83)	9 (9.38)		
University and above	8 (33.33)	76 (79.17)		
**Parity (%)**				0.812^*^
1	19 (79.17)	79 (82.29)		
2	5 (20.83)	16 (16.67)		
3	0 (0.00)	1 (1.04)		
**Delivery method (%)**			0.01	0.926
Natural birth	14 (58.33)	57 (59.38)		
Cesarean section	10 (41.67)	39 (40.63)		
**Preterm birth (%)**			4.90	**0.027**
Yes	5 (20.83)	6 (9.38)		
No	19 (79.17)	90 (93.75)		

^*^Fisher’s exact test. The t-test was used to compare all continuous variables and the results were presented in mean ± SD. The χ2 test or fisher’s exact test was used to compare the categorical variables and the results were presented as frequency and percentage (%). Only variables significant at *p* < 0.05 were entered into univariate conditional logistic regression

We compared the anthropometric measurement between the case and control groups. In girls, maternal height (t = 6.84, *p* =  < 0.001), paternal height (t = 3.91, *p* =  < 0.001), and Maternal history of early puberty (χ^2 =^ 4.11, *p* = 0.043) were significant differences between the case and control groups. In boys, only paternal height (t = 2.88, *p* = 0.002) was significant difference between the case and control groups (Table S1).

The comparison of dietary habits between the case and control groups revealed null significant differences in girls. However, in boys, the feeding method after birth (χ^2^ = 7.17, *p* = 0.028) was significant differences between the case and control groups (Table S2).

Table 2 shows the comparison of sleep habits between the case and control groups. In girls, bedtime at night (t = 3.09, *p* = 0.001), sleep hours per day (t = 4.44, *p* =  < 0.001), night sleep (t = 4.47, *p* =  < 0.001), and wake-up at night (*p* = 0.005, Fisher’s exact test) were significant differences between the case and control groups. In boys, sleep hours per day (t = 3.52, *p* =  < 0.001), and night sleep (t = 2.29, *p* = 0.012) were significant differences between the case and control groups.

**Table 2:** Comparison of sleep habits between the case and control groups

Variables	Case	Control	t /χ^2^	*P*-value
**Girls**				
Bedtime at night (PM)	9.65 ± 0.56	9.85 ± 0.76	3.09	**0.001**
Sleep hours per day(24 h)	9.00 ± 0.76	9.37 ± 0.91	4.44	** < 0.001**
Day sleep	0.27 ± 0.54	0.34 ± 0.70	1.14	0.128
Night sleep	8.73 ± 0.65	9.03 ± 0.69	4.47	** < 0.001**
**Wakeup at night (%)**				0.005^*^
Once	30 (14.93)	51 (25.37)		
2 times	11 (5.47)	2 (1.00)		
3 times	3 (1.49)	1 (0.50)		
4 times	-	-		
5- times	-	-		
Not at all	157 (78.11)	147 (73.13)		
**Time to put a child to sleep at night (%)**				0.311^*^
0–10 min	63 (31.34)	60 (29.85)		
10–20 min	75 (37.31)	64 (31.84)		
20–30 min	37 (18.41)	51 (25.37)		
30–40 min	13 (6.47)	13 (6.47)		
40–60 min	11 (5.47)	7 (3.48)		
> 60 min	2 (1.00)	6 (2.99)		
**Child snore (%)**			6.33	0.097
Never	50 (24.88)	69 (34.33)		
Only on cold or allergy	55 (27.36)	59 (29.35)		
Sometimes	90 (44.78)	68 (33.83)		
Always	6 (2.99)	5 (2.49)		
**Boys**				
Bedtime at night	9.76 ± 0.58	9.86 ± 0.89	0.53	0.298
Sleep hours per day (24 h)	8.82 ± 0.70	9.34 ± 0.63	3.52	** < 0.001**
Day sleep	0.13 ± 0.42	0.28 ± 0.51	1.37	0.087
Night sleep	8.70 ± 0.59	9.06 ± 0.72	2.29	**0.012**
**Wake-up at night (%)**				0.175^*^
Once	11(45.83)	23 (23.96)		
2 times	0 (0.00)	2 (2.08)		
3 times	0 (0.00)	4 (4.17)		
4 times	-	-		
5 times	-	-		
Not at all	13 (54.17)	67 (69.79)		
**Time to put a child to sleep at night (%)**				0.077^*^
0–10 min	13 (54.17)	26 (27.08)		
10–20 min	6 (25.00)	35 (36.46)		
20–30 min	3 (12.50)	23 (23.96)		
30–40 min	1 (4.17)	11 (11.46)		
40–60 min	1 (4.17)	1 (1.04)		
> 60 min	-	-		
**Child snore (%)**				0.376^*^
Never	7 (29.17)	24 (25.00)		
Only on cold or allergy	4 (16.67)	31 (32.29)		
Sometimes	11 (45.83)	37 (38.54)		
Always	2 (8.33)	4 (4.17)		

, no data; ^*^Fisher’s exact test. The t-test was used to compare all continuous variables and the results were presented in mean (± SD). The χ^2^ test or fisher’s exact test was used to compare the categorical variables and the results were presented as frequency and percentage (%). Only variables significant at* p* < 0.05 were entered into univariate conditional logistic regression

Table 3 shows parenting habits between the case and control groups. In girls, Child’s time stared on screening devices (*p* = 0.006, Fisher’s exact test), child’s time spent outdoors (*p* =  < 0.001, Fisher’s exact test), and exposure to secondhand smoke (χ^2^ = 29.13, *p* =  < 0.001) were significant differences between the case and control groups. In boys, Outdoor activities (*p* = 0.021, Fisher’s exact test) and exposure to secondhand smoke (*p* = 0.001, Fisher’s exact test) were significant differences between the case and control groups.

**Table 3:** Comparison of parenting habits between the case and control groups

Variables	Case	Control	χ^2^	*P*-value
**Girls**				
** Child’s time stare on screening devices (%)**				0.006^*^
Never	23 (11.44)	24 (11.94)		
< 30 min	95 (47.26)	69 (34.33)		
30–60 min	48 (23.88)	77 (38.31)		
1–2 h	24 (11.94)	27 (13.43)		
> 2 h	11 (5.47)	4 (1.99)		
**Child’s time spent outdoors (%)**				< 0.001^*^
None	0 (0.00)	2 (1.00)		
Rarely	47 (23.38)	17 (8.46)		
Often but < 1 h	90 (44.78)	96 (47.76)		
1–3 h	52 (25.87)	82 (40.80)		
> 3 h	12 (5.97)	4 (1.99)		
**Outdoor activities (%)**			0.37	0.830
High	48 (23.88)	52 (25.87)		
Moderate	118 (58.71)	112 (55.72)		
Low	35 (17.41)	37 (18.41)		
**Secondhand smoke (%)**			29.13	** < 0.001**
None	97 (48.26)	148 (73.63)		
Several times a month but less than once a week	50 (24.88)	22 (10.95)		
Several times a week but less than once a day	14 (6.97)	12 (5.97)		
Almost everyday	40 (19.90)	19 (9.45)		
**Boys**				
** Child’s time spend on screen stare (%)**				0.230^*^
Never	7 (29.17)	16 (16.67)		
< 30 min	7 (29.17)	41 (42.71)		
30–60 min	5 (20.83)	29 (30.21)		
1–2 h	5 (20.83)	9 (9.38)		
> 2 h	0 (0.00)	1 (1.04)		
**Time spend outdoors (%)**				0.119^*^
None	-	-		
Rarely	4 (16.67)	4 (4.17)		
Often but < 1 h	8 (33.33)	46 (47.79)		
1–3 h	12 (50.00)	43 (44.79)		
> 3 h	0 (0.00)	3 (3.13)		
**Outdoor activities (%)**				0.021^*^
High	11 (45.83)	35 (36.46)		
Moderate	12 (50.00)	33 (34.38)		
Low	1 (4.17)	28 (29.17)		
**Secondhand smoke (%)**				0.001^*^
None	8 (33.33)	69 (71.88)		
Several times a month but less than once a week	9 (37.50)	10 (10.42)		
Several times a week but less than once a day	4 (16.67)	8 (8.33)		
Almost everyday	3 (12.50)	9 (9.38)		

, no data; ^*^Fisher’s exact test. The χ2 test or fisher’s exact test was used to compare the categorical variables, the results were presented as frequency and percentage (%). Only variables significant at *p* < 0.05 were entered into conditional logistic regression

All the variables with significant differences between the case and control groups at *p* < 0.05 from girls or boys were incorporated in the univariate conditional logistic regression analyses, except resident, caregiver’s education, child’s time spent outdoors, and wake-up at night, which were not entered in the univariate conditional logistic regression due to various reasons including collinearity, non-convergence of the model or few responses. Although, child BMI, maternal BMI, and paternal BMI were not significant differences between the case and control groups were exceptionally included in the univariate conditional logistic regression analysis because were considered potential risk factors for precocious puberty in children.

For girls, in the univariate regression, cesarean section (OR = 1.80, 95% CI: 1.19, 2.70), child BMI (OR = 1.17, 95% CI: 1.07, 1.28), Maternal BMI (OR = 1.20, 95% CI: 1.11, 1.31), Maternal history of early puberty (OR = 2.78, 95%CI:1.08, 7.13), formula feeding after birth (OR = 2.19, 95% CI: 1.14, 4.24), and exposure to secondhand smoke several times a month but less than once a week (OR = 3.44, 95% CI: 1.91, 6.22), and almost every day ( OR = 3.46, 95%, CI: 1.78, 6.71) were associated with the increased odds of precocious puberty in girls. On the other hand, maternal height (OR = 0.86 95% CI: 0.81, 0.90), paternal height (OR = 0.92, 95% CI: 0.88, 0.96), bedtime at night (OR = 0.62, 95% CI: 0.45, 0.85), sleep hours (OR = 0.56, 95% CI: 0.43, 0.74), and average night sleep (OR = 0.51, 95% CI: 0.37, 0.70) were associated with decreased odds of precocious puberty in girls (Table 4). In the multivariate model, we included all the variables that were identified to be statistically significant risk factors for precocious puberty from the univariate regressions. We found that cesarean section (OR = 1.99, 95% CI: 1.05, 3.76), child BMI (OR = 1.25, 95% CI: 1.10, 1.43), maternal BMI (OR = 1.13, 95%CI: 1.01, 1.26), and exposure to secondhand smoke several times a month but less than once a week (OR = 4.09, 95%CI: 1.79,9.35), and s almost every day (OR = 6.48, 95% CI: 2.14, 19.56) were consistently associated with the increased odds of precocious puberty in girls. Meanwhile, maternal height (OR = 0.82, 95% CI: 0.75, 0.88), paternal height (OR = 0.91, 95% CI: 0.85, 0.98), bedtime at night (OR = 0.30, 95% CI: 0.17, 0.51), and average night sleep (OR = 0.43, 95% CI: 0.21, 0.86) were associated with the decreased odds of precocious puberty in girls (Table 4).

**Table 4:** Conditional logistic regression for precocious puberty in girls and related factors

Variable	Univariate	*P*-value	Multivariate	*P*-value
	**OR (95%CI)**		**OR (95% CI)**	
**Delivery method**				
Natural birth	1		1	
Cesarean section	1.80 (1.19, 2.70)	**0.005**	1.99 (1.05, 3.76)	**0.034**
**Preterm birth**				
No	1		1	
Yes	1.37 (0.72, 2.58)	0.337	1.44 (0.48, 4.28)	0.513
**Anthropometric measurements**				
Maternal height	0.86 (0.81, 0.90)	** < 0.001**	0.82 (0.75, 0.88)	** < 0.001**
Paternal height	0.92 (0.88, 0.96)	** < 0.001**	0.91 (0.85, 0.98)	**0.012**
Child BMI	1.17 (1.07, 1.28)	** < 0.001**	1.25 (1.10, 1.43)	**0.001**
Maternal BMI	1.20 (1.11, 1.31)	** < 0.001**	1.13 (1.01, 1.26)	**0.036**
Paternal BMI	1.06 (0.98, 1.15)	0.179	-	-
**Maternal history of early puberty**				
No	1		1	
Yes	2.78 (1.08, 7.13)	**0.034**	1.62 (0.42, 6.22)	0.485
**Feeding method after birth**				
Breastfeeding	1		1	
Formula feeding	2.19 (1.14, 4.24)	**0.019**	1.86 (0.62, 5.65)	0.270
Mixed feeding	1.14 (0.71, 1.82)	0.589	0.87 (0.42, 1.77)	0.694
**Sleep habits**				
Bedtime at night	0.62 (0.45, 0.85)	**0.004**	0.30 (0.17, 0.51)	** < 0.001**
Sleep hours	0.56 (0.43, 0.74)	** < 0.001**	0.56 (0.30, 1.07)	0.078
Day sleep	0.83 (0.60, 1.15)	0.256	-	-
Night sleep	0.51 (0.37, 0.70)	** < 0.001**	0.43 (0.21, 0.86)	**0.017**
**Secondhand smoke**				
None	1		1	
Several times a month but less than once a week	3.44 (1.91, 6.22)	** < 0.001**	4.09 (1.79, 9.35)	**0.001**
Several times a week but less than once a day	1.70 (0.72, 4.03)	0.224	0.89 (0.26, 3.07)	0.856
Almost everyday	3.46 (1.78, 6.71)	** < 0.001**	6.48 (2.14, 19.56)	**0.001**
**Child’s time stare on screening devices**				
Never	1			
< 30 min	1.57 (0.79, 3.15)	0.200		
30–60 min	0.70 (0.35, 1.39)	0.310		
1–2 h	0.99 (0.42, 2.33)	0.977		
> 2 h	3.79 (0.93, 5.37)	0.062		
**Outdoor activities**				
High	1			
Moderate	1.14 (0.71, 1.84)	0.584		
Low	1.02 (0.54, 1.94)	0.941		

-, no data; *NA* not available indicating that the variables were omitted due to strong correlation with other variables, *OR* odds ratio, *CI* confidence interval, *BMI* body mass index; *p* < 0.05

For boys, in the univariate model, preterm birth (OR = 4.77, 95%CI: 1.11, 20.65) and exposure to secondhand smoke several times a month but less than once a week (OR = 9.80, 95% CI: 2.51, 38.25) and almost every day (OR = 3.03, 95%CI: 0.68, 13.77) were associated with increased odds of precocious puberty in boys. While paternal height (OR = 0.90, 95%CI: 0.83, 0.97) and sleep hours (OR = 0.31, 95% CI: 0.15, 0.66) were associated with the decreased odds of precocious puberty in boys (Table 5). In the multivariate model, only exposure to secondhand smoke several times a month but less than once a week (OR = 7.94, 95% CI: 1.25, 50.33) was consistently associated with increased odds of precocious puberty in boys (Table 5).

**Table 5:** Conditional logistic regression for precocious puberty in boys and related factors

Variable	Univariate	*P*-value	Multivariate	*P*-value
	**OR (95%CI)**		**OR (95% CI)**	
**Delivery method**				
Natural birth	1		1	
Cesarean section	1.04 (0.42, 2.59)	0.926	0.60 (0.14, 2.54)	0.491
**Preterm birth**				
No	1		1	
Yes	4.77 (1.11, 20.65)	**0.037**	5.22 (0.44, 62.62)	0.192
**Anthropometric measurements**				
Maternal height	0.93 (0.84, 1.02)	0.118	0.95 (0.81,1.11)	0.532
Paternal height	0.90 (0.83, 0.97)	**0.009**	0.93 (0.82, 1.05)	0.243
Child BMI	1.14 (1.00, 1.30)	0.058	1.10 (0.87, 1.40)	0.424
Maternal BMI	1.05 (0.90, 1.22)	0.558	1.12 (0.84, 1.49)	0.430
Paternal BMI	1.09 (0.94, 1.26)	0.268	-	-
**Maternal history of early puberty**				
No	1		1	
Yes	NA	NA	NA	NA
**Feeding method after birth**				
Breastfeeding	1		1	
Formula feeding	3.88 (0.96, 15.57)	0.056	0.97 (0.12, 8.12)	0.996
Mixed feeding	0.67 (0.22, 2.00)	0.471	0.44 (0.08, 2.52)	0.354
**Sleep habits**				
Bedtime at night	0.87 (0.51, 1.49)	0.611	0.59 (0.24, 1.46)	0.254
Sleep hours	0.31 (0.15, 0.66)	**0.003**	0.32 (0.05, 2.25)	0.253
Day sleep	0.42 (0.11, 1.56)	0.195	NA	
Night sleep	0.51 (0.28, 0.93)	**0.028**	0.80 (0.14, 4.60)	0.801
**Secondhand smoke**				
None	1		1	
Several times a month but less than once a week	9.80 (2.51, 38.25)	**0.001**	7.94 (1.25, 50.33)	**0.028**
Several times a week but less than once a day	4.88 (1.07, 22.24)	**0.041**	2.83 (0.45, 17.64)	0.265
Almost every day	3.03 (0.68, 13.77)	0.151	0.99 (0.10, 10.09)	0.996
**Child’s time stare on screening devices**				
Never	1			
< 30 min	0.42 (0.12, 1.49)	0.178		
30–60 min	0.41 (0.11, 1.48)	0.172		
1–2 h	1.21 (0.25, 5.90)	0.816		
> 2 h	NA	NA		
**Outdoor activities**				
High	1			
Moderate	1.29 (0.48, 3.45)	0.611		
Low	0.13 (0.02, 1.02)	0.052		

-, no data; *NA* not available indicating that the variables were omitted due to strong correlation with other variables, *OR* odds ratio, *CI* confidence interval, *BMI* body mass index; *p* < 0.05

**Fig. 2:**
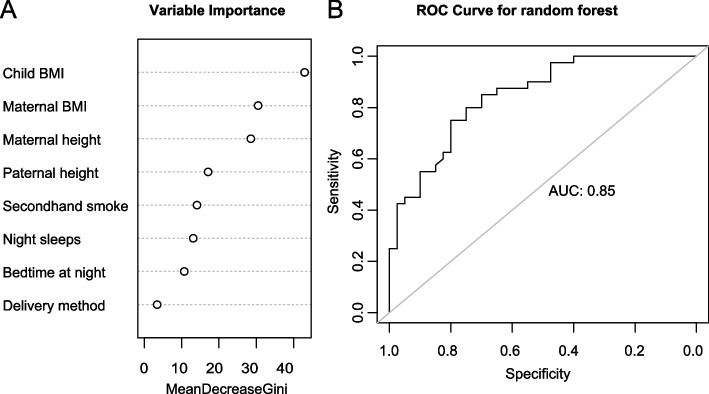
**A**) Variable importance on precocious puberty in girls. **B**) Receiver operating characteristic (ROC) curve was implemented for predictors validation (Area under the curve, AUC = 0.85)

We performed a random forest algorithm a machine learning method to assess the level of variable importance among the risk factors for precocious puberty development in girls. In the random forest model, we used a train set incorporating all the risk factors that were statistically significant from the multivariate conditional logistic regression including cesarean section, child BMI, maternal BMI, secondhand smoke, maternal height, paternal height, bedtime at night, and average night sleep. Moreover, we used a test set to create the ROC-AUC to evaluate the strength of the model. We found that child BMI was the most important risk factor for precocious puberty among 8 factors included in the model (Fig. 2A). The AUC of the model was 0.85 (Fig. 2B), indicating the strength of the model.

## Discussion

In this matched case–control study, we explored the effect of environmental factors on precocious puberty in girls and boys. In the multivariate conditional logistic regression, we found that cesarean section, child BMI, maternal BMI, and exposure to secondhand smoke several times a month but less than once a week and almost every day were risk factors for precocious puberty in girls. While maternal height, paternal height, bedtime at night, and average night sleep were associated with decreased risk of precocious puberty in girls. In boys, only secondhand smoke exposure several times a month but less than once a week was a risk factor for precocious puberty. Moreover, among all risk factors identified, child BMI was the most important risk factor for precocious puberty in girls.

In the present study, 201 girls had precocious puberty (case group) at the mean age of 7.20 ± 1.11 years, and the other 201 normal healthy girls (control group) their mean age was 7.42 ± 0.49 years. In boys, 24 boys had precocious puberty at a mean age of 8.58 ± 2.41 years and other 96 normal healthy boys their mean age was 7.47 ± 0.60 years. Although some of these children, particularly boys their age were older than the corresponding diagnostic criteria at the time of diagnosis, the sexual characteristics of these children appeared before the age of 8 years and 9 years old in girls and boys, respectively. That is, these children came to the clinic long after the onset of secondary sexual characteristics.

We matched cases and controls by age stratified by gender at the ratio of 1:1 and 1:4 for girls and boys, respectively. Our results were similar to the previous studies that girls aged 7 years were more like to develop precocious puberty compared to boys of the same age [4], and also boys were less like to develop precocious puberty compared to girls [4, 25].

In previous studies, childhood BMI [26, 27] and maternal pre-pregnancy obesity [17] were associated with precocious puberty onset. Similarly, in our study, we observed that maternal BMI and child BMI were associated with precocious puberty in girls and not boys in both the univariate and multivariate models. Indeed the effect of maternal BMI and child BMI on precocious puberty onset in girls could be due to the adiposity-puberty timing relationship being stronger and more consistent in girls compared to boys [27].

In this study, cesarean section was an independent risk factor for increased precocious puberty in girls in both the univariate and multivariate models. These associations, however, were not present in boys. On the contrary, a study by Aris et al. reported that cesarean section was associated with pubertal development in boys and not girls [14]. The relatively small study population of boys included in our study or ethnic population heterogeneity may cause this difference. Despite sex variation, the most important information from these two studies is that cesarean section may independently be associated with precocious puberty in children. The mechanism for these associations is yet to be determined. However, previous studies have suggested that cesarean section is related to childhood BMI z-score [28] or higher risk for overweight and obesity [29], through its effects on the gut microbiome development [30] and by an epigenetic mechanism that induces metabolic changes in a child [31]. Consequently, pre-pubertal child weight is associated with early pubertal onset [26, 27]. The association between child BMI and early pubertal was also observed in our study.

Furthermore, in our study, we found that exposure to secondhand smoke whether several times a month but less than once a week or almost every day was consistently associated with increased odds of precocious puberty in girls in both the univariate and multivariate models. While in boys, only exposure to secondhand smoke several times a month but less than once a week was consistently associated with increased odds of precocious puberty in both the univariate and multivariate models. Regardless of secondhand smoke exposure type, the present study is in agreement with another study conducted among South Korean girls, which documented that secondhand smoke was associated with early puberty onset in children [18]. It has been reported that tobacco smoke comprises hundreds of chemicals that could be endocrine disruptors and reproductive toxicants, which could also cause reproductive maturation [32].

Previous studies have reported that maternal history of early puberty [33] and preterm birth were associated with precocious puberty in both girls and boys [13], while formula feeding [15] was associated with precocious puberty in girls. In our study, formula feeding and maternal history of early puberty were associated with early puberty in girls, and preterm birth was associated with precocious puberty in boys in the univariate models. These associations, however, were not present in the multivariate models, indicating that the associations seen may be due to the presence of unmeasured confounders. Likewise, other prior studies have reported a null significant association between formula feeding [34] and preterm birth [22] with precocious puberty in children.

In this study, we also found that maternal height and paternal height were consistently associated with decreased odds of precocious puberty in girls in both the univariate and multivariate models. In boys, only paternal height was associated with decreased precocious puberty, but the association was not present in the multivariate model. These findings indicated that maternal height and paternal height were the factors associated with decreased odds of precocious puberty among girls only. Another study in agreement with our findings reported that parental heights such as maternal and paternal heights are indicators of parental early socioeconomic status, dietary and infections exposure, and have been linked to later pubertal development, particularly age at menarche in girls [35].

Moreover, in the present study, we found that bedtime at night, night sleep, and sleep hours were associated with decreased odds of precocious puberty in girls in the univariate model. However, in the multivariate model, bedtime at night and night sleep was consistently associated with decreased odds of precocious puberty in girls. In boys, night sleep and sleep hours were associated with decreased precocious puberty; however, these associations were not present in the multivariate model. The preceding study reported that delayed sleep and disrupted sleep were associated with precocious puberty [19]. In addition, Hoyt et al. have also documented that shorter sleep duration from later bedtime is associated with earlier pubertal timing among black girls [36]. Another study conducted in China reported similar findings but for a different population, the Chinese ethnicity [37]. In this study, the bedtime of the girls without precocious puberty was at 9.85 ± 0.76 PM and the average night sleep was (9.03 ± 0.69 h.), indicating that they had adequate sleep duration per night compared to those with precocious puberty. From these results, we can suggest that adequate sleep duration per night was a protective factor for precocious puberty development.

Notably, in our study, among eight factors identified to be associated with precocious puberty, child BMI was the most important factor contributing to precocious puberty in girls. Studies have shown that during the lockdown due to the COVID-19 pandemic, unhealthy lifestyles and behaviors were observed including lack of control around food, over-eating or skipping meals, sleep problems, decreased physical activity [7–9], and increased use of screening devices, smoking behavior, and alcohol consumption [9]. All of these could have led to a change in body weight.

The strength of this study includes; firstly, we matched the cases and controls by age stratified by gender at the ratio of 1:1 for girls and 1:4 for boys to account for confounders and selection bias. Secondly, to ensure the quality of clinical research practice, the investigators were trained before the data collection process. Thirdly, we identified the factors, which were associated with precocious puberty in children. Then we selected the most important risk factor for precocious puberty development in girls.

This study has also some limitations. Firstly, most of the information including children’s dietary habits, sleep habits, parenting habits, and parental anthropometric measurements were obtained from children’s caregivers through interview questionnaires and not by a direct measure, which could lead to recall bias. Secondly, considering the relatively small study population of boys included in our study, caution should be taken in comparing the findings for boys and girls. In addition, although the age difference between the case and control groups is not large, this age difference may have a critical issue on the inclusion criteria, considering the diagnostic criteria for precocious puberty. This may have an impact on our findings. Thirdly, our study population is not a national representative. Thus, our findings may not be generalized to all populations of Chinese children. A nation-based case–control study is required to substantiate our findings and generalize the results.

## Conclusions

Our results suggest that environmental factors such as cesarean section, child BMI, and maternal BMI were risk factors for precocious puberty in girls. Moreover, Secondhand smoke was a risk factor for precocious puberty in both girls and boys. Meanwhile, maternal height, paternal height, and adequate sleep duration per night were protective factors for precocious puberty in girls. Of the factors, child BMI was the most important risk factor for precocious puberty. These findings are important as could help to improve the public health strategies for the prevention and control of precocious puberty onset in children.

## Supplementary Information

**Additional file 1:** **Table S1.** Comparison of the anthropometric measures between the case and control groups. **Table S2.** Comparison of Dietary habits between the case and control groups.

## Data Availability

The datasets used in this study are available from the corresponding author upon reasonable request.

## Abbreviations

AUC: Area under the curve
BMI: Body mass index
COVID-19: Coronavirus disease-19
HPA: Hypothalamic–pituitary–adrenal
ROC: Receiver operating characteristic
OR: Odds ratio
SD: Standard deviation
USA: United State of America
