# Supplementary material for: The effect of environmental factors on precocious puberty in children: a case–control study
Source: BMC Pediatr. 2023 May 1;23:207. doi: 10.1186/s12887-023-04013-1 (PMC10149633; doi:10.1186/s12887-023-04013-1)
Supplement: Supplementary file 1 — Additional file 1: Table S1. Comparison of the anthropometric measures between the case and control groups. Table S2. Comparison of Dietary habits between the case and control groups. [file 12887_2023_4013_MOESM1_ESM.docx]

**Supplementary Material**

**Table S1**: Comparison of the anthropometric measure between the case and control groups

| **Variable** | **case** | **control** | **t /ꭓ^2^** | **p-value** |
| --- | --- | --- | --- | --- |
| **Girls** |  |  |  |  |
| Child Height (cm) | 131.34 ± 9.15 | 129.05 ± 5.68 | -3.01 | 0.999 |
| Child Weight (kg) | 29.12 ± 5.76 | 26.34 ± 5.11 | -5.14 | 1.000 |
| Child BMI (kg/m^2^) | 16.80 ± 2.70 | 15.77 ± 2.73 | -3.79 | 1.000 |
| Maternal Height (cm) | 159.56 ± 4.88 | 162.73 ± 4.38 | 6.84 | **<0.001** |
| Maternal Weight (kg) | 57.15 ± 8.21 | 56.11 ± 6.92 | -1.37 | 0.914 |
| Maternal BMI (kg/m^2^) | 22.41 ± 2.75 | 21.18 ± 2.39 | -4.78 | 1.000 |
| Paternal Height (cm) | 173.31 ± 4.69 | 175.24 ± 5.21 | 3.91 | **<0.001** |
| Paternal Weight (kg) | 75.26 ± 9.93 | 75.85 ± 9.86 | 0.60 | 0.275 |
| Paternal BMI (kg/m^2^) | 25.00 ± 2.67 | 24.83 ± 2.75 | -1.21 | 0.887 |
| **Maternal history of early puberty (%)** |  |  | 4.11 | **0.043** |
| Yes | 18 (8.96) | 8 (3.98) |  |  |
| No | 183 (91.04) | 193 (96.02) |  |  |
| **Paternal history of early puberty (%)** |  |  |  | 0.623* |
| Yes | 1 (0.50) | 3 (1.49) |  |  |
| No | 200 (99.50) | 198 (98.51) |  |  |
| **Boys** |  |  |  |  |
| Child Height (cm) | 142.52 ± 9.94 | 131.40 ± 6.037 | -6.99 | 1.000 |
| Child Weight (kg) | 37.01 ± 13.21 | 28.38 ± 5.66 | -4.89 | 1.000 |
| Child BMI (kg/m^2^) | 17.87 ± 4.20 | 16.38 ± 2.70 | -2.13 | 0.982 |
| Maternal Height (cm) | 160.18 ± 6.48 | 161.89 ± 4.02 | 1.63 | 0.053 |
| Maternal Weight (kg) | 55.35 ± 6.87 | 55.66 ± 7.45 | 0.19 | 0.426 |
| Maternal BMI (kg/m^2^) | 21.62 ± 2.82 | 21.24 ± 2.78 | -0.59 | 0.721 |
| Paternal Height (cm) | 170.96 ± 6.87 | 174.87 ± 5.72 | 2.88 | **0.002** |
| Paternal Weight (kg) | 74.71 ± 12.27 | 75.71 ± 9.12 | 0.45 | 0.328 |
| Paternal BMI (kg/m^2^) | 25.50 ± 3.40 | 24.76 ± 2.72 | -1.14 | 0.872 |
| **Maternal history of early puberty (%)** |  |  |  | 1.000* |
| Yes | 0 (0.00) | 2 (2.08) |  |  |
| No | 24 (100.00) | 94 (97.92) |  |  |
| **Paternal history of early puberty (%)** |  |  |  | 1.000* |
| Yes | 0 (0.00) | 1 (1.04) |  |  |
| No | 24 (100.00) | 95 (98.96) |  |  |
| Note: *, Fisher’s exact test. The t-test was used to compare all continuous variables and the results were presented in mean (±SD). The ꭓ^2^ test or fisher’s exact test was used to compare the categorical variables and the results were presented as frequency and percentage (%). Only variables significant at *p* < 0.05 were entered into conditional logistic regressions except for child BMI, maternal BMI and paternal BMI, which entered as potential risk factors for precocious puberty. | | | | |

**Table S2**: Comparison of Dietary habits between the case and control groups

| **Variables** | **Case** | **Control** | **ꭓ^2^** | **P-value** |
| --- | --- | --- | --- | --- |
| **Girls** |  |  |  |  |
| **Feeding method after birth (%)** |  |  | 4.68 | 0.096 |
| Breastfeeding | 71 (35.32) | 83 (41.29) |  |  |
| Formula feeding | 43 (21.39) | 27 (13.43) |  |  |
| Mixed feeding | 87 (43.28) | 91 (45.27) |  |  |
| **Barbecued food (%)** |  |  |  |  |
| Yes | 94 (46.77) | 106 (52.74) | 1.43 | 0.231 |
| No | 107 (53.23) | 95 (47.260 |  |  |
| **Dissert food (%)** |  |  | 0.09 | 0.762 |
| Yes | 175 (87.06) | 177 (88.06) |  |  |
| No | 26 (12.94) | 24 (11.94) |  |  |
| **Soft drinks (%)** |  |  |  |  |
| Yes | 121 (60.20) | 111 (55.22) | 1.02 | 0.313 |
| No | 80 (39.80) | 90 (44.78) |  |  |
| **Vitamin D (%)** |  |  | 0.41 | 0.521 |
| Yes | 134 (66.67) | 140 (69.65) |  |  |
| No | 67 (33.66) | 61 (30.35) |  |  |
| **Child’s bowl materials (%)** |  |  |  | 0.240* |
| Glass | 2 (1.00) | 6 (2.99) |  |  |
| Hard transparent plastics | 3 (1.49) | 0 (0.00) |  |  |
| Hard opaque plastics | 5 (2.49) | 4 (1.99) |  |  |
| Ceramics | 159 (79.10) | 164 (81.59) |  |  |
| Stainless steel | 31 (15.42) | 24 (11.94) |  |  |
| Others | 1 (0.50) | 3 (1.49) |  |  |
| **Boys** |  |  |  |  |
| **Feeding method after birth (%)** |  |  | 7.17 | **0.028** |
| Breastfeeding | 10 (41.67) | 41 (42.71) |  |  |
| Formula feeding | 7 (29.17) | 9 (9.38) |  |  |
| Mixed feeding | 7 (29.17) | 46 (47.92) |  |  |
| **Barbecued food (%)** |  |  | 0.14 | 0.712 |
| Yes | 13 (54.17) | 56 (58.33) |  |  |
| No | 11 (45.83) | 40 (41.67) |  |  |
| **Dissert food (%)** |  |  | 0.13 | 0.723 |
| Yes | 19 (79.17) | 79 (82.29) |  |  |
| No | 5 (20.83) | 17 (17.71) |  |  |
| **Soft drinks (%)** |  |  | 0.08 | 0.784 |
| Yes | 12 (50.00) | 51 (53.13) |  |  |
| No | 12 (50.00) | 45 (46.88) |  |  |
| **Vitamin D (%)** |  |  | 0.32 | 0.573 |
| Yes | 16 (66.67) | 58 (60.42) |  |  |
| No | 8 (33.33) | 38 (39.58) |  |  |
| **Child’s bowl materials (%)** |  |  |  | 0.676* |
| Glass | 1 (4.17) | 7 (7.29) |  |  |
| Hard transparent plastics | - | - |  |  |
| Hard opaque plastics | 1 (4.17) | 1 (1.04) |  |  |
| Ceramics | 20 (83.33) | 80 (83.33) |  |  |
| Stainless steel | 2 (8.33) | 8 (8.33) |  |  |
| Others | - | - |  |  |

Note: -, no data; *, Fisher’s exact test. The ꭓ^2^ test or fisher’s exact test was used to compare the categorical variables and the results were presented as frequency and percentage (%). Only variables significant at p < 0.05 were entered into univariate conditional logistic regressions.
